# Population norms and cut-off-points for suboptimal health related quality of life in two generic measures for adolescents: the Spanish VSP-A and KINDL-R

**DOI:** 10.1186/1477-7525-7-35

**Published:** 2009-04-21

**Authors:** Vicky Serra-Sutton, Montse Ferrer, Luis Rajmil, Cristian Tebé, Marie-Claude Simeoni, Ulrike Ravens-Sieberer

**Affiliations:** 1 Agència d'Avaluació de Tecnologia i Recerca Mèdiques, Barcelona, Spain; 2 CIBER Epidemiología y Salud Pública, CIBERESP, Spain; 3 Institut Municipal d'Investigació Mèdica, Barcelona, Spain; 4 Service de Santé Publique. EA 3279. Faculté de Médicine, Marseille, France; 5 Building W 29 (Erikahaus), University Clinic Hamburg-Eppendorf, Hamburg, Germany

## Abstract

**Background:**

Health-related quality of life (HRQL) outcome measures are complex and for further application in clinical practice and health service research the meaning of their scorings should be studied in depth. The aim of this study was to increase the interpretability of the Spanish VSP-A and KINDL-R scores.

**Methods:**

A representative sample of adolescents aged 12 to 18 years old was selected in Spain. The Spanish VSP-A and KINDL-R, two generic HRQL measures (range: 0–100), were self-administered along with other external anchor measures (Strengths and Difficulties Questionnaire, Oslo Social Support Scale and self-declaration of chronic conditions) and sent by post. Percentiles of both HRQL questionnaires were obtained by gender, and age group and effect sizes (ES) were calculated. Receiver Operating Characteristic curves and related sensitivity (SE) and specificity (SP) values were also computed.

**Results:**

The Spanish VSP-A and KINDL-R were completed by 555 adolescents. A moderate ES was shown in Psychological well-being between younger and older girls (ES: 0.77) in the VSP-A and small ES in the KINDL (ES: 0.41) between these groups. A SE and SP value close to 0.70 was associated to a global HRQL score of 65 in the VSP-A and 70 in the KINDL-R, when compared to anchors measuring mental and psychosocial health. Adolescents with scores bellow these cut-off points showed a moderate probability of presenting more impairment in their HRQL.

**Conclusion:**

The results of this study will be of help to interpret the VSP-A AND KINDL-R questionnaires by comparing with the general population and also provide cut-off points to define adolescents with health problems.

## Background

Measurement of health related quality of life (HRQL) started in the 70's as a complement to traditional clinical outcomes. As a consequence of the increase in the survival of chronic conditions and life expectancy, healthcare has gone beyond the cure of illness. These facts and also a greater implication of patients in clinical decision making, have lead to the use of more subjective outcomes to measure the effectiveness of treatments such as HRQL [1]. The most extended definition of this outcome presents a multidimensional perspective that includes patients or populations' points of view of their health, and also the influence on their ability to deal with daily activities considered important for individuals [2]. Many health-related quality of life (HRQL) measures have been developed for children and adolescents in the last decade. However, their use is still limited and restricted mainly to research areas. To generalize their application in different contexts, interpretation has been identified as one of the main barriers.

Several authors have argued [3,4], that scores of HRQL by themselves are difficult to interpret. Thus, interpretability implies the degree to which one can assign easily understood meaning to an instrument's quantitative score. There are different approaches to facilitate the interpretability of HRQL scores; one of the most used for generic questionnaires is the application of normative values from general populations [5]. This strategy allows comparing individuals or groups of patients with the distribution of scores, and help putting into context an individual or group score by comparing it with a corresponding reference group [6]. Furthermore, we are also interested in defining cut-off points for groups with more impaired HRQL. Generally, the selection is made in terms of the distribution deciding which proportion of the population would be in the group of ill health. For example, half of the population if we select the median, or the 30% with worst scores in the case of a percentile 30. However, it is an arbitrary decision and a meaning is not provided to a given cut-off point. For this purpose, independent well-known indicators could help to select the most appropriate HRQL cut-off point and could provide a direct health meaning. For this anchor strategy, different indicators such as mortality or disease diagnoses have been used [7]. This strategy has been commonly used to interpret scores of mental health scales such in the case of the Chid Behaviour Checklist (CBCL) [8] or the Strengths and Difficulties Questionnaire (SDQ) [9] to differentiate an ill mental health status from a healthy one. Nevertheless, few studies have addressed these issues of interpretability using HRQL questionnaires in child or adolescent populations.

Finally, the simultaneous comparison of questionnaires also has proved to be of use in gaining interpretation of their scorings [10]. Issues such as the content, nature of development and cultural context of the HRQL measures could make scores differ for similar domains. The Spanish versions of the French *Vecú Santé Perçue de l'Adolescent *(VSP-A) and the German Questionnaire for measuring health-related quality of life in children and adolescents (KINDL), two generic HRQL questionnaires, were adapted in parallel and were included in the Kidscreen project as validation instruments [11]. As part of the process of obtaining the Spanish versions, interpretability issues were defined. The aims of this study were to facilitate the interpretation of the Spanish VSP-A and KINDL-R by obtaining general population based reference norms and identifying appropriate cut-off points to define probabilities of worse HRQL compared to external anchors. A simultaneous comparison of the results of both measures was also carried out.

## Methods

### Design and sample selection

This study was based on a cross-sectional descriptive telephone and postal survey carried out simultaneously in 13 European countries in the context of the Kidscreen project. The first stage of sample selection was carried out by telephone using the random digital dialling technique. A sample size of 1800 children and adolescents per country was considered necessary to detect a minimally important difference of half a standard deviation (SD) in HRQL scores, and it has been described in more detail elsewhere [12]. The sample frame was all households with a fixed telephone line and with children and adolescents aged 8–18. For comparison reasons between questionnaires, only the sub-sample of adolescents was included in the present study. Data protection requirements of the European Parliament were followed and checked by the European Commission (Directive 95/46/EC of the European Parliament). Parents consent was received to participate in the mail survey. If parents and adolescents agreed to participate when contacted by telephone, a postal survey was sent to them. A sample of 577 adolescents completed a self-administered survey at home and sent it back to research team by post. The fieldwork was carried out between April 2003 and November 2003. The postal response rate was 44.9% in the case of Spain and 555 cases were finally included in this study. A percentage of 3.8 adolescents presented incomplete information or were either younger than 12 or older than 18.

### Description of the instruments and variables

The Spanish versions of the VSP-A and KINDL-R were administered together with other demographic and health status variables.

The VSP-A and the KINDL-R are two generic HRQL measures that were developed in France and Germany respectively [13,14]. The VSP-A was developed for adolescents aged 11 to 17 and includes 39 items distributed in 9 domains: "Vitality", "Physical well being (WB)", "Psychological well being (WB)", "Body image", "Relations with friends", "Relations with parents", "Relations with teachers", "School work", "Leisure", and two additional modules that report information on "Relations with health professionals" and on "Sentimental and sexual life" (these two latter modules were not assessed in this study). In addition to dimension scores, the VSP-A allows the generation of a global score and index of HRQL (12 item version) [15]. The KINDL-R was developed for children and adolescents aged 8–11 (Kid-KINDL) and 12–16 (Kiddo-KINDL). It includes 24 items distributed in 6 domains: "Physical well being (WB)", "Psychological well being (WB)", "Self-esteem", "Family", "Friends" and "School" and additional modules for children and adolescents with chronic conditions (not assessed in this study) and allows creating a global HRQL score. Both HRQL measures include a Likert scale with 5 options in a 4-week and 1-week recall period, respectively. Domain scores include a score range of 0 to 100. Higher scores indicate better HRQL. For comparison reasons, the versions assessed in this study were the Spanish VSP-A and Spanish Kiddo-KINDL-R, administered to adolescents aged 12–18.

#### Other socio-demographic and health variables

Socio-demographic variables were also collected from adolescents such as sex and age (12–15, 16–18 years old) and perceived socio-economic status using the Family Affluence Scale (FAS) [16]. Other variables included the self-declaration of a chronic condition collected from a checklist (yes, no) and the Strengths and Difficulties Questionnaire (SDQ), a brief behavioural screening questionnaire that allows the classification of children and adolescents in healthy, borderline or noticeable mental health groups [9,17]. The Oslo Social Support Scale, which consists of 3 items, was also administered and categorized as strong, moderate and poor social support [18,19]. This scale collects information on the number of people who can provide a sense of security to the adolescent in terms of instrumental and emotional support. These versions were answered by parents in the case of the SDQ and by adolescents in the case of socio-demographical variables, Oslo Scale, and presence of a chronic condition.

### Statistical analysis

Descriptive statistics of the Spanish VSP-A and KINDL-R domain scores were computed.

#### General population based reference values and magnitude of score differences

Reference values of the Spanish VSP-A and KINDL-R were described in a sample of adolescents after stratification by gender and two age groups (12–15 and 16–18 years old) as differences were expected based on finding of the original versions and existing literature [13,14,20]. Mean, standard deviation, and deciles were also calculated. Distribution based approaches are used for the interpretation of HRQL questionnaires and allow to quantify the magnitude of score differences between groups. At cross-sectional level effect sizes (ES) can be computed and described as small (0.2–0.5), moderate (0.51–0.8) or large (> 0.8) values.

#### Use of external anchors

In this study, 3 external anchors were used: the SDQ (categorized as normal versus borderline-noticeable mental health problem), the self-declaration of a chronic condition (yes versus no); and the Oslo Social Support Scale (categorized as poor versus moderate-high). To incorporate the information from these external anchors to the Spanish VSP-A and KINDL-R, their frequencies were computed for each of the 10 points of global HRQL scores in each questionnaire. Receiver Operating Characteristics (ROC) curves and the Area under the Curve (AUC) were computed to assess the discrimination ability of selected VSP-A and KINDL-R scores in relation to these external anchors [21].

Sensitivity (SE) and specificity (SP) values related to an "optimal" HRQL cut-off-point were also described. These values are used in epidemiological studies, and especially in diagnostic tests [22]. A SE value describes the probability that a given measure adequately classifies an "ill" or "exposed" individual, while a SP value describes the probability of measure to correctly classify a person that is not "ill", or is not "exposed" to a given risk [23]. In this study, the cut-off-scores in selected HRQL were chosen according to the highest SE and SP values. Comparable domains in the Spanish VSP-A and KINDL-R were selected for comparison with anchors measuring similar concepts. Psychological well-being domains in the VSP-A and KINDL-R were compared with the SDQ used as an anchor measuring mental health; relations with parents/parents and relations with friends/friends in both HRQL questionnaires were compared to the Oslo Social Support Scale. In the case of the anchor measuring physical chronic conditions, it was compared with domains in the VSP-A and KINDL-R of physical well-being. Finally, all selected anchors were compared with the global HRQL scores in the VSP-A and KINDL-R.

## Results

Most adolescents were 12–15 years old (63.5%) and half of the sample were girls (50.8%), while most declared being from a middle socio-economic background (51.6%), measured by the FAS (Table 1). Three percent of the Spanish adolescent reference sample (n = 555) was classified as suffering a noticeable mental distress measured by SDQ, 10.3% declared a physical chronic condition, and 17.5% of adolescents scored poor social support.

**Table 1:** Description of sample by socio-demographic and health characteristics (n = 555)

**Variables**	**N (%)**
**Sex/age**	
Girls	
*12–15*	177 (62.8)
*16–18*	105 (37.2)
Boys	
*12–15*	177 (64.8)
*16–18*	96 (35.2)
FAS*	
Low	119 (21.6)
Middle	285 (51.6)
High	148 (26.8)
**Chronic condition**	
No	495 (89.7)
Yes	57 (10.3)
Psychiatric or mental health (SDQ)**	
Normal	455 (85.2)
Borderline	62 (11.6)
Clinical	17 (3.2)
**Social support**	
Strong	146 (26.6)
Moderate	306 (55.8)
Poor	96 (17.5)

*FAS: Family Affluence Scale.

** SDQ: Strengths and Difficulties Questionnaire

### Descriptive statistics of the Spanish VSP-A and KINDL-R

More than 50% of adolescents presented scores above or close to 70 in most VSP-A domains, except for "Relations with teachers" and "School work" (Table 2). In the KINDL-R, 50% of adolescents scored above 70 in all domains, except for "School". The percentage of missing values in this study was less than 6% in the Spanish VSP-A domains, and less than 3% in the Spanish KINDL-R. The questionnaires did not present any floor effect. A ceiling effect was observed in the domains "Body Image" and "School Work" in the VSP-A (35.3% and 16.0%, respectively), and also in the "Psychological well-being", "Friends" and "Parents" domains in the KINDL-R (19.5%, 20.8% and 25.4%, respectively).

**Table 2:** Descriptive results of the Spanish VSP-A and KINDL-R domains in a representative sample of non-institutionalized adolescents in Spain (n = 555)

Domains (no. of items)	Mean (SD)*	**Median**	**Missing values**	**Effect **%		**Observed range**	
	
			%	Floor	Ceiling	Min.	Max.
	
**Spanish VSP-A**							
Vitality (5)	70.1 (18.6)	70.0	3.9	0.0	6.1	10.0	100.0
Physical WB** (4)	71.7 (17.4)	75.0	3.8	0.0	6.3	12.5	100.0
Psychological WB** (5)	68.7 (20.1)	70.0	3.8	0.0	6.3	5.0	100.0
Body image (2)	74.7 (27.4)	87.5	3.8	2.6	35.3	0.0	100.0
Relations w. friends (5)	77.5 (17.0)	80.0	5.2	0.0	13.0	20.0	100.0
Relations w. parents (4)	69.8 (20.6)	75.0	3.4	0.0	11.1	6.2	100.0
Relations w. teachers (3)	60.0 (25.3)	58.3	4.3	2.6	11.8	0.0	100.0
School work (2)	60.5 (28.3)	62.5	3.8	6.5	16.0	0.0	100.0
Leisure (4)	69.8 (19.6)	75.0	3.4	0.4	8.0	0.0	100.0
Global score	69.2 (14.1)	69.7	6.3	0.0	0.0	16.2	98.8
	
**Spanish KINDL-R**							
Physical WB** (4)	76.9 (16.9)	81.2	2.7	0.0	11.0	12.5	100.0
Psychological WB** (4)	82.5 (15.3)	87.5	2.7	0.0	19.5	31.5	100.0
Self-esteem (4)	70.7 (20.5)	75.0	2.9	0.2	11.0	0.0	100.0
Friends (4)	82.7 (14.8)	87.5	2.1	0.0	20.8	6.2	100.0
Parents (4)	80.6 (19.3)	87.5	2.7	0.2	25.4	0.0	100.0
School (4)	53.0 (15.8)	50.0	2.9	0.0	0.0	12.5	93.7
Global score	74.5 (12.3)	76.0	2.9	0.0	0.0	28.1	97.9

*SD: standard deviation. **WB: well-being; w: with

### Description of population reference values and the magnitude of score differences

In general, the younger group presented higher (better) scores in all domains of HRQL for both girls and boys (please see Additional file 1 and Additional file 2). A moderate effect size (ES) was shown in domains such as Psychological well-being in the VSP-A between younger and older girls (ES: 0.77), and among older teens, between boys and girls (ES: 0.59). In the Physical well-being domain moderate and large ES were shown (ES: 0.47 between younger and older girls; and ES: 0.81 between boys and girls of 16 to 18 y. old). In the KINDL-R, differences between groups were smaller, showing an ES: 0.41 for differences between older and younger girls in their Psychological well-being, and an ES: 0.34 between older boys and girls in their Physical well-being. These differences should be taken into account when interpreting the normative values as girls tend to present lower scorings.

### Use of external anchors

Table 3 shows the percentage of adolescents with a probable psychosocial or physical chronic condition for each of the 10-point intervals of the Spanish global HRQL scores. Adolescents with a VSP-A global score < 50 were more likely to present a noticeable mental health problem, or a psychosocial or chronic condition (53%, 55% and 16%, respectively). In the case of the KINDL-R, adolescents with a global HRQL score < 50 showed the highest probability of presenting a noticeable mental health problem, a psychosocial problem or chronic condition (67%, 80% and 20% respectively). On the other hand, global HRQL scores near 80 reflect a low probability (11% for VSPA and 16% for KINDL-R) of presenting any of these health problems.

**Table 3:** Perceptual distribution (%) of participants in the Spanish reference sample with a psychosocial or chronic health problem according to their global HRQL score in the Spanish VSP-A and KINDL-R

	total sample	adolescents with a borderline-noticeable mental problem^b^	adolescents with low social support^c^	adolescents with a self-declared chronic condition	adolescents with any of the 3 health problems
					
**Global VSP-A **	N^d^	n (%)	n (%)	n (%)	n (%)
					
0-49.9a	49	25 (53.2)^e^	27 (55.1)	8 (16.3)	37 (75.5)
50–59.9	77	25 (32.9)	22 (29.3)	7 (9.2)	40 (52.0)
60–69.9	140	16 (11.7)	27 (19.4)	11 (7.9)	49 (35.0)
70–79.9	129	9 (7.0)	9 (7.1)	4 (3.1)	21 (16.3)
80–89.9	100	3 (3.0)	5 (5.1)	3 (3.0)	11 (11.0)
90–100	29	0 (0.0)	1 (3.6)	0 (0.0)	1 (3.4)
					
**Global KINDL-R**	N	n (%)	n (%)	n (%)	n (%)
					
0–49.9^a^	15	10 (66.7)	12 (80.0)	3 (20.0)	15 (100.0)
50–59.9	57	24 (42.1)	16 (28.6)	6 (10.5)	33 (58.0)
60–69.9	107	17 (17.3)	26 (25.0)	9 (8.5)	45 (42.0)
70–79.9	167	15 (9.0)	25 (15.2)	9 (5.4)	42 (25.1)
80–89.9	143	7 (5.2)	10 (7.1)	8 (5.6)	23 (16.0)
90–100	54	0 (0.0)	2 (3.8)	1 (1.9)	3 (5.0)

HRQL: health related quality of life.^a^due to small number of cases included in scores between 0 and 49.9 they were re-categorized in one group. ^b^measured with the SDQ. ^c^measured with the Oslo Scale.^d^missing values are presented in some subgroups. ^e ^the percent value of each cell is computed as: n in the cell/N in the whole sample [Example, n:25/N:47 = (53.2). Note 2 missing values in the SDQ, N = 49].

**Figure 1:**
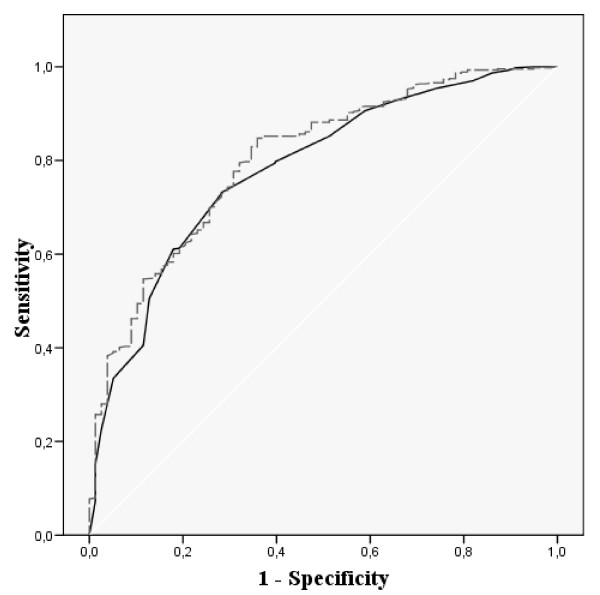
**ROC curves for adolescents' scores in Psychological Well-being and Global scores in the Spanish VSP-A versus a borderline-noticeable mental health problem (measured by SDQ)**. AUC: Area Under Curve; 95% CI: 95% Confidence Interval; SE: Sensitivity; SP: Specificity. [Black line] Psychological well-being AUC: 0.78 (95% CI: 0.73–0.84) Optimal cut-off-point: 61.2. SE: 0.73 SP: 0.72 [Dashed line] Global HRQL score AUC: 0.80 (95% CI: 0.75–0.85) Optimal cut-off-point: 63.7 SE: 0.74 SP: 0.70

**Figure 2:**
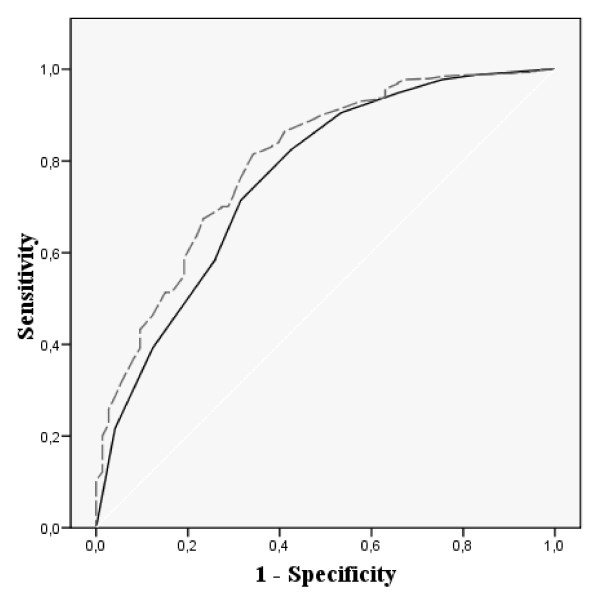
**ROC curves for adolescents' scores in Psychological Well-being and Global scores in the Spanish KINDL-R versus a borderline-noticeable mental health problem (measured by SDQ)**. AUC: Area Under Curve; 95% CI: 95% Confidence Interval; SE: Sensitivity; SP: Specificity. [Black line] Psychological well-being. AUC: 0.76 (95% CI: 0.70–0.83). Optimal cut-off-point: 78.1 SE: 0.71 SP: 0.68. [Dashed line] Global HRQL score. AUC: 0.80 (95% CI: 0.74–0.86). Optimal cut-off-point: 70.1 SE: 0.73 SP: 0.70

**Figure 3:**
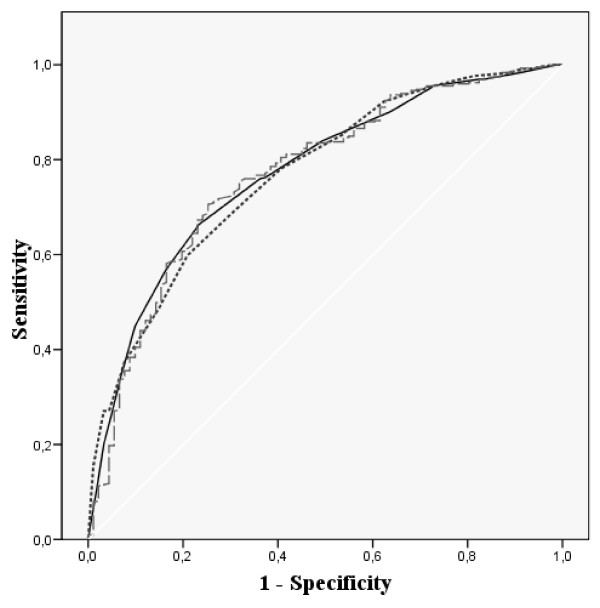
**ROC curves for adolescents' scores in Relations with Friends, Relations with Parents and Global score in the Spanish VSP-A versus poor social support (measured by the Oslo Social Support scale)**. AUC: Area Under Curve; 95% CI: 95% Confidence Interval; SE: Sensitivity; SP: Specificity. [Black line] Relation with parents. AUC: 0.77 (95% CI: 0.72–0.82). Optimal cut-off-point: 60.4 SE: 0.76 SP: 0.64. [Dotted line] Relation with friends. AUC: 0.77 (95% CI: 0.72–0.82). Optimal cut-off-point: 67.5 SE: 0.78 SP: 0.59. [Dashed line] Global HRQL score. AUC: 0.77 (95% CI: 0.71–0.82). Optimal cut-off-point: 64.8 SE: 0.72 SP: 0.72

**Figure 4:**
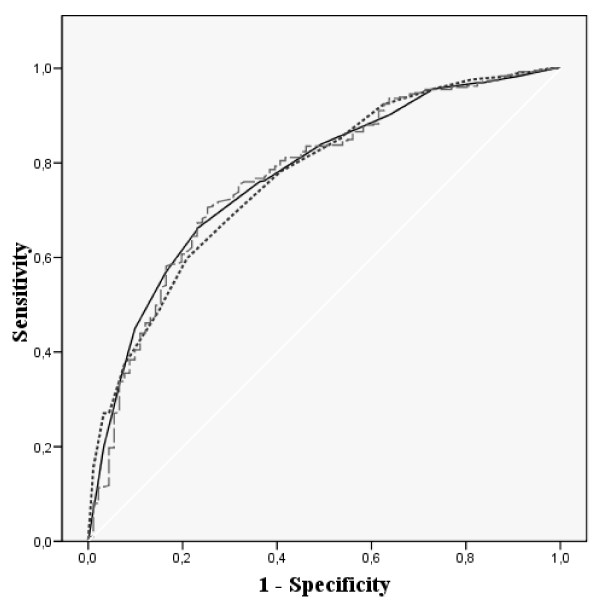
**ROC curves for adolescents' scores in Friends, Parents and Global score in the Spanish KINDL-R versus poor social support (measured by the Oslo Social Support scale)**. AUC: Area Under Curve; 95% CI: 95% Confidence Interval; SE: Sensitivity; SP: Specificity [Black line] Parents AUC: 0.68 (95% CI: 0.62–0.74) Optimal cut-off-point: 71.9 SE: 0.77 SP: 0.52 [Dotted line] Friends AUC: 0.70 (95% CI: 0.63–0.76) Optimal cut-off-point: 78.1 SE: 0.68 SP: 0.62 [Dashed line] Global HRQL score AUC: 0.72 (95% CI: 0.67–0.78) Optimal cut-off-point: 71.3 SE: 0.70 SP: 0.63

Figures 1, 2, 3 and 4 show the ROC curves of the Spanish VSP-A and KINDL-R global and selected domain scores to predict more impaired HRQL. The agreement between "Psychological well-being" and global HRQL scores with a probable borderline-noticeable mental health problem (Figure 1 and Figure 2) was very similar for both questionnaires (AUC around 0.8). The discrimination ability of the domains measuring social HRQL (Figure 3 and Figure 4) such as family and friend relationship domains in the VSP-A and KINDL-R when compared to poor social support (measured by the Oslo scale), showed that the VSP-A presented a higher AUC (0.77) compared to the KINDL-R (range: 0.68 – 0.70). In the case of "Physical well-being" scores in the VSP-A and KINDL-R, and reported chronic condition (data not shown), the AUC were lower and similar for both questionnaires (AUC = 0.63 and 0.64 for VSP-A and KINDL-R, respectively). For global HRQL scores, this agreement was higher for the VSP-A questionnaire (AUC of 0.70 versus 0.60). Regarding the selection of a cut-off point in the Spanish global VSP-A closest to 65.0, a sensitivity value (SE) of 0.72 to 0.74, and specificity value (SP) of 0.70 to 0.72 were shown in relation to a probable noticeable mental health problem or psychosocial health problem. In the Spanish KINDL-R, a global score close to 70.0 was related to a SE value of 0.70 to 0.73, for the screening of a probable mental or psychosocial health problem and a SP associated value between 0.70 and 0.63, respectively.

## Discussion

The results of this study allow increasing the possibilities for interpretation of two generic HRQL questionnaires in future studies where the questionnaires are applied. Interpretation strategies imply more than the assessment of validity; they should potentially include the interpretability of scores for researchers, clinicians, decision makers and patients or general society. A study that included the content analysis of selected generic HRQL measures for these age groups has shown that similar instruments show different contents, even for apparently similar domains such as physical or psychological well-being [24]. In the context of paediatric measures, some research teams have defined the model of the instrument based on literature or expert consensus, and included the opinion of children or adolescents for the definition of item content. This is also the case of the KINDL-R questionnaire [14]. The VSP-A was developed using exclusively the opinion of adolescents for defining item and domain content [13]. The final model included in both questionnaires has been defined through psychometric testing.

Results of psychometric testing of the Spanish versions of the VSP-A and KINDL-R have shown acceptable validity and reliability coefficients [25,26]. Even if some differences were shown regarding discrimination abilities of specific domains in the VSP-A and KINDL-R, there have shown similar values regarding AUC or SE and SP values to detect those adolescents with more impairment in their HRQL. These results imply that both instruments are adequate for describing health needs of adolescents and for their application in health service research in Spain. Moreover, the use of the Spanish VSP-A and KINDL-R in future applied studies will allow continuing the assessment of their validity and longitudinal reproducibility in groups with different clinical or socio-demographical characteristics. The results of the present study will aid potential users of these questionnaires in interpreting the results of their own studies.

General population-based reference norms are the approach used for height and weight in the assessment of paediatric growth and it is also the interpretation strategy most used for generic HRQL. The use of percentiles to describe scores does not require assumptions of normal distributions and makes it possible to interpret the HRQL scores: 1) it provides with information on the amount of HRQL impairment of an individual or a group by comparing the score obtained, with the distribution of scores of the corresponding population; 2) differences in percentile position may help to determine the size of score differences, observed either in a child over time or between two groups or individuals. On the other hand, reference norms could also help to fix therapeutic objectives taking into account that the maximum theoretical score of a questionnaire would not be considered the maximum attainable. In fact, although the VSP-A and the KINDL-R have scores ranging from 0 to 100 (the best HRQL), very few teenagers score this maximum. When interpreting longitudinal changes [7,27], these population reference values also can help to interpret how far scores of patients are before treatment, and if they achieve scores close to the normative values after the intervention.

Differences in age and gender should be taken into account when assessing the meaning of "healthy" or "ill" HRQL scores. Girls in this study have reported lower vitality, physical and psychological well-being and lower scores in general, compared to boys. These results are consistent with the original version results and other studies that have applied HRQL measures in adolescents and also imply that the same score could have different meaning according to the individual or group evaluated. Consistent findings in the literature have presented a gender pattern in favour of boys and younger teens in several international studies [28,29]. These differences could be due to girls presenting more health needs, or even expressing them more openly than boys. The definition of the age groups in the present study has been due to a theoretical perspective following the organization of the Educational and Health System in Spain. Adolescents attend ESO (Secondary Obligatory School) from 12 to 15 years old and then High School from 16 to 18 years old. Moreover, Paediatric services are defined for children and adolescents up to 15 years old. Previous studies in Spain using similar age groups have shown similar results in younger and older adolescents' HRQL scores [20]. Regarding the impact of pubertal changes on HRQL, a study published by the Kidscreen group showed that the most relevant changes when comparing groups aged 8–18 are at the age of 12–13 in relation to younger children, especially in their physical and psychological well-being. From these ages onwards, their HRQL scores worsen gradually [29]. Future studies including greater and longitudinally based samples could help to study the impact of age developmental process on HRQL scores in Spanish children and adolescents using the VSP-A and KINDL-R questionnaires.

The use of cut-off points also help in the interpretation of HRQL scores together with the use of norm values [3,5]. The cut-off points identified by the external anchors for the Spanish VSP-A and KINDL-R, have potentially allowed differentiating 'healthy' groups from groups with more impairment in their HRQL. In other studies that include mental health scales to distinguish a normal score from a psychiatric health problem or other studies of HRQL also include the definition of cut-off points, either based on conceptual and empirical strategies comparing scores with diagnostic groups or using cluster analysis to test the classification of cases in "healthy" or "ill health" [8,30]. In our study, the use of SE and SP values related to a given cut-off point helped to assess how well a test is discriminating between groups [22]. Optimal SE and SP values are those that find the highest value for both estimators. In this study the optimal SE and SP values were associated to a global score close to 65.0 in the VSP-A and close to 70.0 in the KINDL-R and imply that adolescents with these scores show a moderate probability of being in good health.

Limitations of this study should be mentioned. Even if the sample design aimed to obtain a representative, non-institutionalised sample of adolescents in Spain, the sampling method based on a telephone interview and postal survey caused a lower response rate than other administration methods (ex. school based) in the Spanish context. More detail of the representativeness of this sample can be found in another published study of the Kidscreen project [12]. A short phone interview of non-responses was carried out in Spain. As a result, analysis of representativeness showed that, in general, it was acceptable compared to EUROSTAT data regarding age and sex. The lower than expected response rate obtained in this study (45% instead of 70%) has introduced a limitation in the stratification of the sample by each age. This fact has also introduced a probable response bias implying infra-estimations of HRQL scores in the Spanish sample. The use of both instruments in different populations, including clinical samples and from different geographical areas in Spain will allow for continuing the validation and interpretation strategies as recommended in the literature. The application of these measures in clinical samples will be needed to increase the interpretability of scores in ill adolescents compared to the reference values in this study. Longitudinal studies will increase the interpretability of scores of these questionnaires to detect changes over time when a health intervention has been implemented. Finally, even if clinical information could not be obtained and medical conditions were self-reported, the screening measures used in the present study have helped to interpret the scores of HRQL domains setting cut-of-points of adequate health.

The applicability of HRQL measures for children and adolescents are similar for those in adult ages such as the identification of health needs, studies to determine risk factors, assessment of effectiveness and efficacy of health services, monitoring of health at population and clinical level, health planning or priority setting [1,3,31]. Nevertheless, applied studies are much less frequent in these age groups mainly because there has been a process of development and psychometric testing before available measures could be used. Some of the challenges for the measurement of HRQL in paediatric ages imply the use of this outcome to deepen in the knowledge of factors related to better or worse health status or use in clinical practice and public health for the assessment of health interventions.

## Conclusion

The results of this study will be of use for future users of the Spanish VSP-A and KINDL-R questionnaires, especially to assess how close or not scores are from those considered as "healthy" HRQL. Moreover, the increase in the use of HRQL in the evaluation of health-care makes it necessary not only to assess if scores improve after an intervention, but also if they reach similar population reference values.

## Abbreviations

VSP-A: Vecú Santé Perçue de l'Adolescent;KINDL: Questionnaire for measuring health-related quality of life in children and adolescents; HRQL: Health-Related Quality of Life; CBCL: Child Behaviour Checklist; SDQ: Strength and Difficulties Questionnaire; SD: Standard Deviation; ES: Effect Size; ROC: Receiver Operating Characteristic; AUC: Area under the Curve; SE: Sensitivity; SP: Specificity; CI95%: Confidence interval 95%; WB: Well-Being

## Competing interests

The authors declare that they have no competing interests.

## Authors' contributions

VSS, MF contributed to the design of this study, analysed data and co-wrote this paper. LR is the Principal Investigator, contributed to the design of this study and commented on this paper.

CT, MCS and URS commented on this paper. MCS and URS are the original authors of the VSP-A and KINDL-R respectively. URS is also the coordinator of the Kidscreen project.

## Supplementary Material

Additional file 1 **Population reference values of the Spanish VSP-A by age and gender (P: Percentiles. Spain n = 555)**. Additional table Click here for file

Additional file 2 **Population reference values of the Spanish KINDL-R by age and gender (P: Percentiles. Spain n = 555)**. Additional table Click here for file
