# Supplementary material for: Population norms and cut-off-points for suboptimal health related quality of life in two generic measures for adolescents: the Spanish VSP-A and KINDL-R
Source: Health Qual Life Outcomes. 2009 Apr 21;7:35. doi: 10.1186/1477-7525-7-35 (PMC2678997; doi:10.1186/1477-7525-7-35)
Supplement: Additional file 1 — Population reference values of the Spanish VSP-A by age and gender (P: Percentiles. Spain n = 555). Additional table [file 1477-7525-7-35-S1.doc]

**Additional file 1. Population reference values of the Spanish VSP-A by age and gender (P: Percentiles. Spain n=555)**

|  | **Girls (n=282)** | | | | | | | | |  | **Boys (n=273)** | | | | | | | | |
| --- | --- | --- | --- | --- | --- | --- | --- | --- | --- | --- | --- | --- | --- | --- | --- | --- | --- | --- | --- |
|  | VIT | PHY | PSY | BO | FRI | PA | TEA | SCH | LE |  | VIT | PHY | PSY | BO | FRI | PA | TEA | SCH | LE |
| ***12-15 y.old (n=354)***  P10  P20  P30  P40 Median P60  P70  P80  P90  **Mean**  (SD)  ***16-18 y.old (n=201)***  P10  P20  P30  P40 Median P60  P70  P80  P90 Mean (SD) | 45.0  50.0  60.0  65.0  **70.0**  75.0  80.0  85.0  95.0  **69.0**  (19.0)  38.0  50.0  55.0  60.0  **65.0**  70.0  75.0  79.0  85.0  **63.0**  (18.1) | 50.0  56.2  62.5  68.7  **68.7**  75.0  81.2  87.5  93.7  **70.0**  (17.2)  41.2  43.7  50.0  58.7  **62.5**  65.0  75.0  75.0  81.2  **61.8**  (17.5) | 40.0  55.0  65.0  70.0  **75.0**  80.0  85.0  90.0  95.0  **71.1**  (20.0)  28.0  40.0  49.0  50.0  **55.0**  60.0  65.0  70.0  85.0  **55.5**  (20.4) | 12.5  50.0  62.5  75.0  **75.0**  87.5  100.0  100.0  100.0  **69.7**  (31.0)  25.0  37.5  50.0  62.5  **75.0**  75.0  87.5  100.0  100.0  **66.8**  (26.9) | 55.0  65.0  75.0  80.0  **85.0**  85.0  90.0  95.0  100.0  **79.2**  (16.6)  50.0  60.0  65.0  70.0  **77.5**  85.0  90.0  90.0  100.0  **75.6**  (17.7) | 38.1  50.0  56.2  62.5  **75.0**  81.2  87.5  93.7  100.0  **69.7**  (22.2)  42.9  50.0  56.2  62.5  **68.7**  75.0  81.2  87.5  100.0  **68.3**  (20.6) | 33.3  50.0  50.0  58.3  **58.3**  75.0  75.0  83.3  100.0  **63.4**  (25.2)  29.2  33.3  50.0  50.0  **58.3**  66.7  75.0  75.0  95.8  **58.8**  (24.2) | 26.2  50.0  50.0  62.5  **75.0**  75.0  83.7  100.0  100.0  **68.0**  (26.2)  25.0  37.5  50.0  50.0  **62.5**  75.0  75.0  87.5  100.0  **61.3**  (27.7) | 43.7  56.2  62.5  65.0  **68.7**  75.0  81.2  87.5  87.5  **69.0**  (19.5)  31.2  50.0  50.0  62.5  **62.5**  68.7  75.0  81.2  93.7  **63.4**  (21.3) |  | 55.0  61.0  70.0  75.0  **75.0**  80.0  85.0  94.3  100.0  **76.1**  (16.5)  45.0  60.0  65.0  70.0  **70.0**  75.0  80.0  85.0  95.0  **70.4**  (17.3) | 56.2  66.7  68.7  75.0  **81.2**  81.2  87.5  93.7  100.0  **78.0**  (15.1)  56.2  62.5  66.7  68.7  **75.0**  81.2  87.5  87.5  93.7  **75.2**  (15.4) | 55.0  65.0  65.0  70.0  **75.0**  80.0  85.0  90.0  100.0  **76.3**  (15.8)  43.0  55.0  55.0  65.0  **65.0**  70.0  76.0  85.0  95.0  **67.0**  (18.6) | 45.0  62.5  75.0  80.0  **87.5**  100.0  100.0  100.0  100.0  **79.5**  (24.4)  50.0  62.5  75.0  87.5  **87.5**  100.0  100.0  100.0  100.0  **83.9**  (21.2) | 55.5  65.0  70.0  75.0  **80.0**  85.0  90.0  95.0  100.0  **78.5**  (16.6)  52.0  60.0  65.0  70.0  **75.0**  80.0  85.0  95.0  100.0  **75.3**  (17.7) | 43.7  56.2  62.5  68.7  **75.0**  81.2  87.5  87.5  93.7  **72.4**  (18.9)  40.6  50.0  59.4  62.5  **68.7**  75.0  81.2  87.5  100.0  **67.9**  (20.6) | 25.0  41.7  50.0  50.0  **58.3**  75.0  75.0  83.3  100.0  **61.3**  (25.8)  20.0  31.7  41.7  50.0  **50.0**  58.3  66.7  75.0  83.3  **53.4**  (24.5) | 8.75  25.0  50.0  50.0  **62.5**  75.0  75.0  87.5  100.0  **57.1**  (29.6)  12.5  25.0  50.0  50.0  **50.0**  62.5  75.0  75.0  87.5  **53.6**  (26.6) | 50.0  56.2  68.7  75.0  **75.0**  81.2  87.5  93.7  100.0  **75.2**  (18.0)  43.7  50.0  62.5  68.7  **71.9**  75.0  81.2  87.5  93.7  **69.4**  (18.0) |

VIT: Vitality; PHY: Physical well-being; PSY: Psychological well-being; BO: Body-image; FRI: Relations with friends; PA: Relations with parents; TE: Relations with teachers; SCH: School work; LE: Leisure. The median and mean scores are shaded in grey. SD: Standard deviation.
