# Supplementary material for: Population norms and cut-off-points for suboptimal health related quality of life in two generic measures for adolescents: the Spanish VSP-A and KINDL-R
Source: Health Qual Life Outcomes. 2009 Apr 21;7:35. doi: 10.1186/1477-7525-7-35 (PMC2678997; doi:10.1186/1477-7525-7-35)
Supplement: Additional file 2 — Population reference values of the Spanish KINDL-R by age and gender (P: Percentiles. Spain n = 555). Additional table [file 1477-7525-7-35-S2.doc]

**Additional file 2. Population reference values of the Spanish KINDL-R by age and gender (P: Percentiles. Spain n=555)**

|  | **Girls (=282)** | | | | | |  | **Boys (n=273)** | | | | | |
| --- | --- | --- | --- | --- | --- | --- | --- | --- | --- | --- | --- | --- | --- |
|  | PHY | PSY | SEL | FRI | PA | SCH |  | PHY | PSY | SEL | FRI | PA | SCH |
| ***12-15 y.old (n=354)***  P10  P20  P30  P40 Median P60  P70  P80  P90  **Mean**  (SD)  ***16-18 y.old***  ***(n=201)***  P10  P20  P30  P40 Median P60  P70  P80  P90 Mean (SD) | 50.0  62.5  68.7  75.0  **75.0**  81.2  87.5  87.5  93.7  **74.8**  (17.9)  50.0  56.2  62.5  68.7  **68.7**  75.0  81.2  87.5  92.5  **69.9**  (17.0) | 62.5  75.0  75.0  81.2  **87.5**  87.5  93.7  100.0  100.0  **83.5**  (14.6)  51.2  62.5  68.7  68.7  **75.0**  81.2  87.5  91.2  93.7  **75.4**  (15.8) | 37.5  50.0  56.2  68.7  **68.7**  75.0  81.2  87.5  93.7  **68.7**  (21.5)  37.5  50.0  50.0  56.2  **68.7**  68.7  75.0  75.0  87.5  **63.0**  (19.4) | 62.5  68.7  75.0  81.2  **87.5**  87.5  93.7  100.0  100.0  **82.8**  (14.7)  62.5  68.7  75.0  75.0  **75.0**  87.5  87.5  93.7  100.0  **79.1**  (14.5) | 50.0  66.2  75.0  81.2  **87.5**  93.7  93.7  100.0  100.0  **81.3**  (19.7)  50.0  62.5  68.7  75.0  **81.2**  87.5  93.7  100.0  100.0  **78.1**  (22.4) | 37.5  43.7  43.7  50.0  **56.2**  62.5  62.5  68.7  78.1  **56.0**  (16.5)  31.2  37.5  43.7  43.7  **50.0**  50.0  56.2  56.2  62.5  **47.8**  (13.7) |  | 62.5  75.0  75.0  81.2  **87.5**  87.5  93.7  95.0  100.0  **83.1**  (14.0)  56.2  68.7  68.7  75.0  **81.2**  81.2  87.5  93.7  93.7  **77.0**  (16.1) | 68.7  75.0  81.2  87.5  **87.5**  93.7  93.7  100.0  100.0  **86.6**  (13.8)  59.4  62.5  75.0  81.2  **84.4**  87.5  90.6  93.7  100.0  **80.7**  (15.7) | 50.0  62.5  68.7  75.0  **81.2**  87.5  93.7  97.5  100.0  **77.5**  (19.4)  50.0  56.2  62.5  68.7  **68.7**  75.0  81.2  87.5  96.9  **70.8**  (18.0) | 68.7  75.0  81.2  81.2  **87.5**  93.7  93.7  100.0  100.0  **85.9**  (14.2)  59.4  68.7  75.0  75.0  **81.2**  87.5  93.7  93.7  100.0  **80.8**  (15.3) | 56.2  68.7  75.0  81.2  **87.5**  92.5  93.7  100.0  100.0  **82.7**  (17.0)  52.5  62.5  68.7  75.0  **81.2**  87.5  93.7  100.0  100.0  **78.2**  (18.6) | 37.5  43.7  43.7  50.0  **56.2**  56.2  62.5  68.7  75.0  **55.3**  (15.8)  31.2  37.5  38.7  43.7  **50.0**  50.0  56.2  62.5  66.2  **48.8**  (14.1) |

PHY: Physical well-being; PSY: Psychological well-being; SEL: Self-esteem; FRI: Friends; PA: Parents; TE: Teachers;

SCH: School. The median and mean scores are shaded in grey. SD: Standard deviation.
